# FPGA-Based Smart Sensor for Drought Stress Detection in Tomato Plants Using Novel Physiological Variables and Discrete Wavelet Transform

**DOI:** 10.3390/s141018650

**Published:** 2014-10-09

**Authors:** Carlos Duarte-Galvan, Rene de J. Romero-Troncoso, Irineo Torres-Pacheco, Ramon G. Guevara-Gonzalez, Arturo A. Fernandez-Jaramillo, Luis M. Contreras-Medina, Roberto V. Carrillo-Serrano, Jesus R. Millan-Almaraz

**Affiliations:** 1 CA Ingeniería de Biosistemas, División de Investigación y Posgrado, Facultad de Ingeniería, Universidad Autónoma de Querétaro, Cerro de las Campanas s/n, Querétaro 76010, Qro., Mexico; E-Mails: cduarte20@alumnos.uaq.mx (C.D.-G.); irineo.torres@uaq.mx (I.T.-P.); ramon.guevara@uaq.mx (R.G.G.-G.); aafernandez@hspdigital.org (A.A.F.-J.); mcontreras@hspdigital.org (L.M.C.-M.); 2 HSPdigital-CA Telemática, DICIS, Universidad de Guanajuato, Carr. Salamanca-Valle km 3.5+1.8, Palo Blanco, Salamanca 36885, Gto, Mexico; E-Mail: troncoso@hspdigital.org; 3 División de Investigación y Posgrado, Facultad de Ingeniería, Universidad Autónoma de Querétaro, Cerro de las Campanas s/n, Querétaro 76010, Qro., Mexico; E-Mail: roberto.carrillo@uaq.mx; 4 Facultad de Ciencias Físico-Matemáticas, Universidad Autónoma de Sinaloa, Av. De las Américas y Blvd. Universitario, Cd. Universitaria, Culiacán 80000, Sinaloa, Mexico

**Keywords:** drought detection, smart sensor, transpiration dynamic, photosynthesis measurement, plant water stress monitoring

## Abstract

Soil drought represents one of the most dangerous stresses for plants. It impacts the yield and quality of crops, and if it remains undetected for a long time, the entire crop could be lost. However, for some plants a certain amount of drought stress improves specific characteristics. In such cases, a device capable of detecting and quantifying the impact of drought stress in plants is desirable. This article focuses on testing if the monitoring of physiological process through a gas exchange methodology provides enough information to detect drought stress conditions in plants. The experiment consists of using a set of smart sensors based on Field Programmable Gate Arrays (FPGAs) to monitor a group of plants under controlled drought conditions. The main objective was to use different digital signal processing techniques such as the Discrete Wavelet Transform (DWT) to explore the response of plant physiological processes to drought. Also, an index-based methodology was utilized to compensate the spatial variation inside the greenhouse. As a result, differences between treatments were determined to be independent of climate variations inside the greenhouse. Finally, after using the DWT as digital filter, results demonstrated that the proposed system is capable to reject high frequency noise and to detect drought conditions.

## 1. Introduction

Plant stress is any factor that promotes unfavorable growing conditions on plants. Soil drought is an environmental stress that affects crop productivity more than any other factor. Current monitoring devices for precision agriculture usually take into account climatic variables. However, it is desirable to have tools that provide information about plant health in order to explore responses under unfavorable conditions.

The main responses of plants under drought are photosynthetic dysfunction and overproduction of Reactive Oxygen Species (ROS) that are highly reactive and deteriorate the normal plant metabolism through oxidative damage of plant macromolecules [1]. These effects are cumulative; depend on the crop growth stage and the severity and frequency of the drought event. Fortunately, plants have several resistance mechanisms to survive under drought conditions; these range going from morphological to biochemical adaptations at subcellular, cellular, and organ level [2]. The disadvantage of such survival strategies is that they rely on limited plant development and low yield. However, the study of those mechanisms allows the development of strategies to increase drought tolerance without losing productivity, for example: crop varieties associated with high yield can be targeted in breeding programs to induce drought tolerance. Biotechnology research has made it possible to identify and change drought-responsive genes inducing some desired qualitative and quantitative traits. Finally, the exogenous application of plant growth regulators (PGR) have proven to enhance drought tolerance in plants [3]. Concluding, the impact of drought on agricultural practices and the requirements to maintain a constant improvement of drought resistant varieties makes the development of technological tools to detect and monitor drought in plants imperative.

Different methodologies have been proposed for early detection of drought stress in plants. The predominant tendency is to use thermography and hyperspectral vision [4]. Other methods use impendence, thermal or gas exchange principles. The thermography utilizes infrared thermometer sensors or thermal cameras to measure the canopy temperature (*T_c_*) and to define crop water stress indexes [5,6]. However, *T_c_* measurement presents low resolution and it is susceptible to meteorological conditions and foliage geometric structure such as leaf angles [7]. On the other hand, hyperspectral analysis consists of monitoring changes in the chlorophyll fluorescence or in photochemical reflectance. The problem with chlorophyll fluorescence analysis is that it requires a dark chamber to isolate a plant sample [8,9]. In this manner, the chlorophyll fluorescence response may occur as variations in magnitude or phase [4,10]. Monitoring reflectance has been applied to study entire crops; several wavelengths have been explored to find better responses and relations with current state of the crops. 705–750 nm was determined to be a suitable wavelength range to be used to explore plant response to water stress [11]. The aforementioned results have been supported by many researchers who have proposed different indexes to detect and even measure the effects of drought [12,13]. Though, the performance of hyperspectral imaging is critically affected by ambient illumination changes [11], it requires successive monitoring of plants [14], the image acquisition is complicated where drones or satellites are required [15,16].

Limitations to identify small variations in water stress could be solved using plant-based sensors. In this manner a simple sensor mounted on the leaf could measure variations in the temperature gradient according to the water content of the plant [17]. Electrical impedance spectroscopy is robust to environmental noise and has higher sensitivity than hyperspectral imaging; it has been proven to detect water stress, even environmental changes and nutrient deficit. However, additional studies are necessary to understand the environmental effects on plant impedance [18,19].

Gas exchange systems constitute the basis of most photosynthesis measurement tools. This consists of using Infrared Gas Analyzer (IRGA)-based carbon dioxide (CO_2_) sensors to measure the difference between ambient CO_2_ concentration and the concentration in a transparent chamber where a plant leaf is isolated [20]. These tools also estimate important phenomena such as transpiration and stomatal conductance [21,22]. Despite the fact that CO_2_ exchange method is more sensitive than fluorescence techniques to environmental changes; a higher amount of information related to plant physiology can be obtained [23].

The objective of this article is the development of a novel smart sensor that performs a new signal processing methodology to minimize the noise in a photosynthesis measurement system which is based on CO_2_ exchange method. Furthermore, the proposed system is utilized to detect and monitor the effects of soil drought in tomato plants. The signal processing methodology combines average decimation and Kalman filters to improve signal quality, and an additional filtering stage based on Discrete Wavelet Transform (DWT) to explore plants signal response. Therefore, short and long-term novel indexes were proposed to provide a set of information regarding the response of plants to drought.

The smart sensor was implemented in a FPGA due to its parallel computation capabilities and flexible configurability. It made possible to implement the aforementioned algorithms to calculate *in-situ* and in *real-time* the physiological processes of plants for decision making, data storing and off-line processing purposes. In order to validate the drought detection capabilities of the developed smart sensor, an experimental setup was carried out using tomato plants in a greenhouse. Because of this, three smart sensors controlled by a coordinator were installed to monitor specific groups of plants subjected to induced drought conditions. Finally, interesting relations between drought and plant physiological responses were obtained.

## 2. Background

### 2.1. Plant Transpiration, Photosynthesis Dynamics and Drought

Photosynthesis and transpiration are two of main physiological processes in plants. Photosynthesis is a process performed by plants and other organisms to convert light into chemical energy that can later be released to fuel the organism activities. More specifically, light energy drives the synthesis of carbohydrates from carbon dioxide and water with the generation of oxygen (O_2_). On the other hand, transpiration is an important component of temperature regulation because plants can dissipate the heat input from sunlight through phase exchange of water that escape into the atmosphere. This process controls the water movement through the plant and the evaporation from aerial parts, especially from the leaves [24]. Leaf surfaces contain pores called stomata; the aperture of these pores is conducted by guard cells. Through the stomata, plants exchange moisture with the atmosphere and permit the diffusion of CO_2_; transpiration also changes osmotic pressure of cells and enables the flow of mineral nutrients and water from roots to shoots. Since both processes share the same pathways, carbon assimilation carries a loss of water to the atmosphere through the stomata. Consequently, effects of drought over both physiological processes are closely related with parameters that have been previously stated [25].

Plant responses to soil drought can change according to the severity and frequency of the stress and the effects over physiological process does not occur immediately and linearly. Therefore, the severity of the stress and plant responses to drought can be summarized in three phases. Phase 1: Mild water stress. A reduction in transpiration is caused by a decline of stomatal conductance (*g_s_*) is presented [26]. However, the rate of net CO_2_ assimilation remains constant because stomatal closure inhibits transpiration more than it decreases intercellular CO_2_ concentrations. Even during early stages of drought stress, the plant increases its water-use efficiency. Phase 2: Moderate water stress. Here, a further decrease of *g_s_* is accompanied by large decrease of mesophyll conductance (*g_m_*), and a small but significant decrease in photosynthetic activity appears [27]. Finally, in phase 3: Severe water stress. Stomatal conductance drops below its threshold value, the photosynthetic capacity is impaired, and a permanent damage of photosystems suggests that the leaves are enduring oxidative stress, senescence and remobilization of leaf nutrients [28]. At this point, the effects of drought are irreversible and are reflected in the net CO_2_ assimilation of the plant [29].

Plants response is often affected by different stress conditions. Because of this, monitoring of multiple plant related variables promises to be a more accurate tool to assess the real plant state. Furthermore, changes on stomatal conductance and transpiration are more specifically related to soil water content than leaf water content. Consequently, stomata related changes are far more significant than changes in net photosynthesis; that could be considered for early detection. However, drought stress eventually provokes irreversible damage in photosystems and plant efficiency which allows utilizing this variable as a long-term indicator, principally after water recovery.

### 2.2. Estimation of Plant Physiological Processes

As aforementioned, the basis of the gas exchange method for photosynthesis (*P_n_*) estimation involves a comparison between CO_2_ concentration in the atmosphere (*C_i_*) and CO_2_ concentration in the leaf chamber (*C_o_*) where the plant sample is isolated. Additionally, it is necessary to estimate the mass flow rate per leaf area (*W*) as stated in its equation in Table 1 [30]. Here, *P* is the atmospheric pressure in Bar, *V* is the volumetric air flow in liters per minute (lpm), *T_a_K* is air temperature in Kelvin (K) and *A* is leaf area in cm^2^. The 2005.39 constant is an adjusted coefficient to change mass units to mol, surface to m^2^ and time from minutes to seconds. In a similar manner, estimation of transpiration (*E*) is performed, but in this case by measuring the H_2_O vapor exchange. Other important processes such as stomatal conductance, vapor pressure deficit (*VPD*) and leaf to air temperature difference (*LATD*) can be estimated by using equations that have been previously stated by many authors and are summarized in Table 1 [22,25,31].

## 3. Smart Sensor

**Figure 1.**
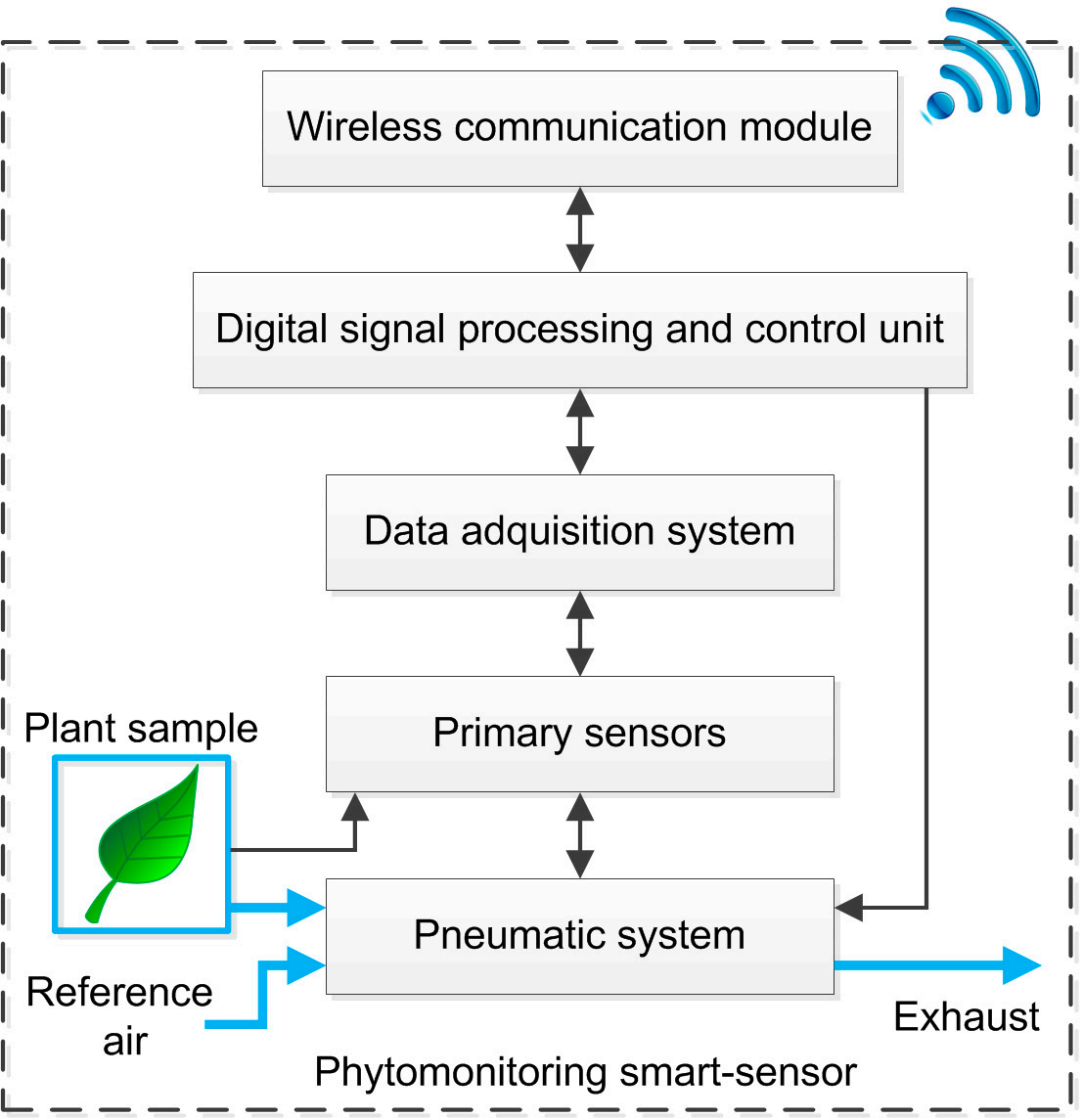
Block diagram of the phytomonitoring smart-sensor.

The proposed smart sensor fuses a water vapor and a CO_2_ gas exchange system into the same pneumatic line in order to estimate *P_n_* and *E*. The system also estimates other phenomena such as *g_s_*, *VPD*, and *LATD*. Climatic variables such as solar radiation, temperature and relative humidity can also be monitored with the same hardware. As can be seen in Figure 1, five stages (black blocks) integrate the smart-sensor.

**Figure 2.**
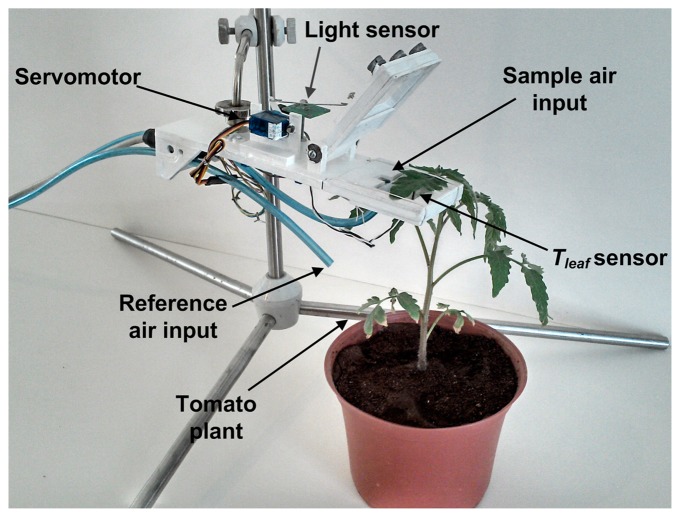
Leaf chamber and sensors arrangement.

First, the pneumatic system uses a transparent acrylic chamber to isolate the plant sample; a set of electrovalves switching between the environment air reference or leaf chamber, and an air pump applies negative pressure in order to move air through the pneumatic system where the sensors are attached. The set of primary sensors are located in two places as can be seen in Figure 2. A Honeywell Pt1000 Resistance Temperature Detector (RTD) configured to measure in a range from 0 to 65 °C with a measurement error of ±0.3 °C is located in the leaf chamber, which has a suitable range to monitor leaf temperature of the plant on contact. Also, an OSRAM SFH5711 ambient solar radiation sensor with a 0 to 100,000 lux range and measurement error of ±0.04% of its measured value is located near the plant sample, which is isolated in the leaf chamber. In the rest of the pneumatic system are attached a Sensirion SHT75 digital Micro Electro Mechanical System (MEMS)-based sensor that measures temperature and relative humidity (*RH*) of air with a resolution of 14-bits for temperature and 12-bits for *RH* and measurement error of ±0.4 °C and ±1.8%, respectively. An OMRON DF6 MEMS-based flow sensor is used to monitor the volumetric flow of the pneumatic line; this sensor has a measurement range of 0 to 5 lpm with measurement error of ±0.4%. To monitor the atmospheric pressure, a Freescale Semiconductor MPX4115A absolute pressure sensor with a range of 15 to 115 kPa and a measurement error of ±1.5% was utilized. Finally, in order to monitor the CO_2_ concentration an Edinburgh Instruments Gascheck 2 IRGA based CO_2_ sensor is required. The sensor has a measurement range of 0–3000 ppm with measurement error of ±30 ppm.

The signals of all primary sensors are standardized to a 0 to 5 V output format by using OpAmp-based modules. Then, each sensor reading is entered into the Data Acquisition System (DAS) through an analog front end, 2nd order anti-alias low pass filter with a cut-off frequency of 20 Hz. A 12-bit Analog to Digital Converter Texas Instruments ADS7844 sampled the previously filtered sensor signals. The ADS7844 communicates via SPI with the third stage, the Digital Signal Processing and Control Unit (DSPCU) which is embedded in a low-cost EP2C35F672C6 FPGA that manages the ADS7844 at 200 kg samples per second (ksps), and also communicates via a 2-wire serial interface with the digital SHT75 sensor. This FPGA-processor is also responsible for controlling the mechanism used in the pneumatic line. The aforementioned tasks are performed simultaneously because of the parallel capabilities of the FPGA. Moreover, this unit performs data filtering operations in order to improve the quality of signals. Finally, the DSPCU estimates and transmits the physiological processes together with environmental readings to a coordinator device by using a wireless communication module.

### Digital Signal Processing Techniques

**Figure 3.**
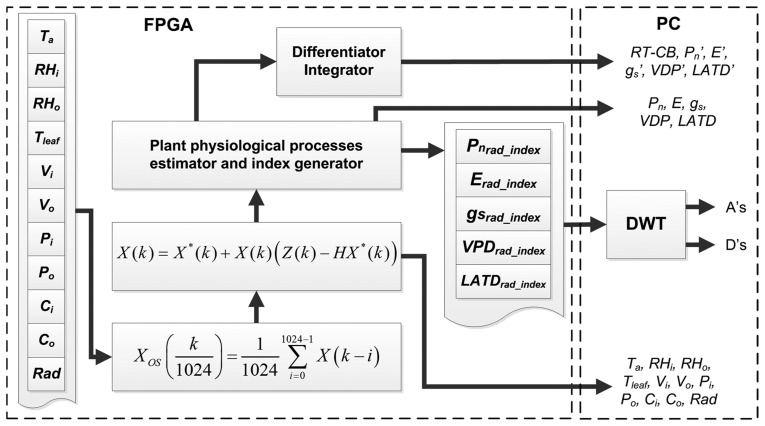
FPGA filtering stage and plant physiological estimator unit.

Because the experiment is performed in a noisy environment where the greenhouse microclimate presents sudden changes due to the influence of external weather, two stages of signal processing units are embedded inside the FPGA in order to reduce the amount of noise in primary sensors readings. As is illustrated in Figure 3, previously the estimation of plant processes, the signals *X*(*k*) from the primary sensors pass through a 1024th order average decimation filter, where a single average sample reduced in quantization and undesirable noise is obtained every second. Furthermore, the oversampled versions of sensor readings *X_os_*(*k*) are introduced into the Kalman filters to obtain new filtered signals *X_osk_*(*k*) [32].

As can be seen in Figure 3, once all the *X_osk_*(*k*) are calculated, the plant physiological process estimator computes *P_n_*, *E*, *g_s_*, *VPD*, and *LATD* from primary sensors readings. In addition, the proposed smart sensor provides a new version of the aforementioned process, in which spatial variations induced for the solar radiation can be reduced by using the simple index expressed in Equation (1). Herein, *X_norm_*(*k*) represents the normalized version of the signals *P_n_*, *E*, *g_s_*, *VDP* or *LATD*. Meanwhile *Rad_norm_*(*k*) is the normalized version of radiation, but considers the maximum *Rad* value from all nodes. Finally, *X_rad_index_*(*k*) is the index that relates the physiological process to the radiation at the time when the sample was acquired:

(1) $$X_{\text{rad}\_\text{index}}(k)=\frac{X_{\text{norm}}(k)}{Rad_{\text{norm}}(k)}$$

Moreover, the plant physiological estimator unit calculates the first derivative of *P_n_′*, *E′*, *g_s_′*, *VPD′*, and *LATD′* in order to explore phenomena involved in the changes of physiological activity. This task is performed by using a discrete derivative as described in Equation (2), which can easily be implemented in the FPGA. Herein, *X′*(*k*) can represent any of the physiological processes previously estimated:

(2) $$X^{\prime }(k)=\frac{X(k)-X(k-1)}{T_{s}}$$

This unit also computes the Real Time-Carbon Balance (*RT*-*CB*), by integrating *P_n_*, this index, as was previously reported in [22], describes the accumulation of carbon due to the photosynthesis activity. It is calculated by using Equation (3), which is the discrete time version of the integral:

(3) $$RT-CB=T_{s}\sum_{k=0}^{N}P_{n}(k)$$

Furthermore, these signals are transmitted to a PC together with data from primary sensors to be stored and plotted. In addition, the PC performs a DWT to *X_rad_index_*(*k*) signals in order to explore the responses at different frequencies.

## 4. Experimentation and Results

### 4.1. Experimental Setup

**Figure 4.**
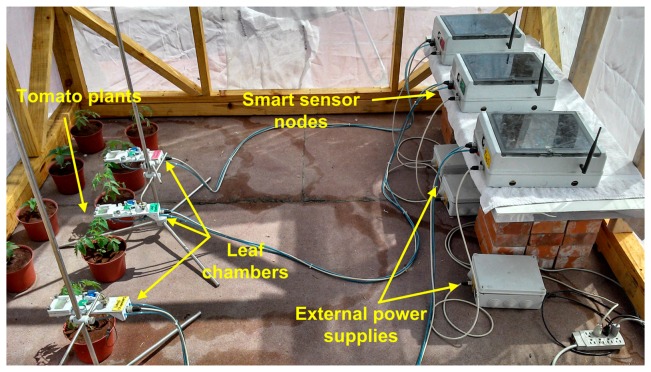
Experimental setup for smart sensors under real operating conditions.

The experiment illustrated in Figure 4 was conducted during 2013 in a research greenhouse located at an altitude of 54 m, in the Universidad Autonoma de Sinaloa, School of Biology, Culiacan Rosales, Sinaloa, Mexico (24°48′0″N, 107°23′0″W). The greenhouse was a single span arch type with 30 m^2^ of ground, equipped with a commercial climate controller. The plants used for the experiment were single genotype tomatoes (*Solanum lycopersicum* L.) variety Raffaello; it is an indeterminate tomato appropriate for cultivation within greenhouse conditions and it is resistant to pests and diseases.

The variation factor in the experiment was the content of water in the soil at two levels: (a) The reference that represents plants irrigated at field capacity and (b) The drought treatment where the irrigation is recurrently suspended one day in order to reach water deficit in the soil. Three smart sensor nodes were used to measure the responses of plants to different irrigation levels. In addition, three tensiometers Irrometer model R were installed in the monitored plants in order to monitor the content of water in the soil. These sensors have a 0 to 100 kPa range with an accuracy rating of ±2%.

### 4.2. Sample Preparation

The tomato plants were germinated and transplanted into two liters containers where the plants were grown in greenhouse conditions until they were an appropriate size to attach the sample in the leaf chambers. The substrate used was a volcanic stone called tezontle, screened to homogenize the particles diameter and ensure same soil conditions (apparent density of 605 kg·m^−3^). In order to avoid other variation factors, all the plants were irrigated with Steiner solution at a concentration according to the plant growth stage. Finally, in order to obtain reliable responses between different plants, it was necessary to standardize a method to select the leaves that would be monitored. The selected leaves were located at the same height, not to low leaves because it is reported that they are the first to lose the photosynthetic activity due to aging, and not the top leaves because they are the last to respond to drought [33].

### 4.3. In-Situ Node Adjustment and Validation

**Figure 5.**
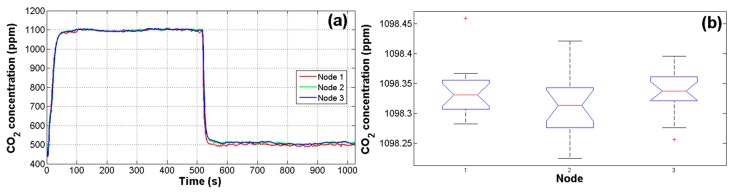
Validation test of IRGA CO_2_ sensors. (**a**) 1 cycle monitoring with an 1100 ppm CO_2_ reference; (**b**) Analysis of variance boxplot results.

Due to the high noise in CO_2_ signals and the fact that reliability of estimations depends on measurements of primary sensors, a test was performed in order to assess the responses of the IRGA CO_2_ sensors using an 1100 ppm CO_2_ reference. As can be seen in Figure 5a, the first 512 samples correspond to the CO_2_ reference and the next 512 samples correspond to the inside air of the closed greenhouse without plants. This test was performed for 16 cycles for the three nodes. Finally, with the average of cycles, an ANOVA was carried out to evaluate the response of nodes. The value of alpha *α* for this and other tests in this work was set to 0.05 (95% of confidence). The resulting *p*-value of the analysis was 0.2205, which is higher than 0.05 that represents the upper boundary considered for statistical differences between treatments [34]. In this manner, the resulting *p*-value represents no significant difference between node readings. As it can be observed in Figure 5b there are only two outlier data points in node 1 and 3. The means in the analysis were 1098.4, 1098.3, and 1098.3 ppm with standard deviations of 4.49, 4.31, and 4.67 ppm respectively for nodes 1, 2, and 3.

### 4.4. Filtering Results

**Figure 6.**
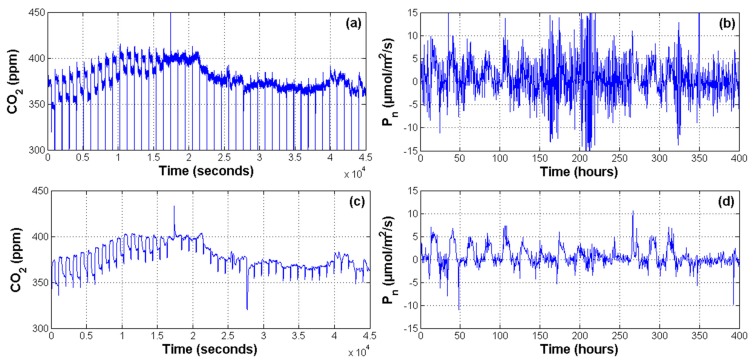
Digital filtering results over photosynthesis estimation. (**a**) Non-filtered CO_2_ signal; (**b**) *P_n_* estimation based on raw signals; (**c**) filtered CO_2_ signal; and (**d**) *P_n_* estimation based on filtered signals.

Figure 6 illustrates improvements over signal quality after the filtering stages. In the Figure 6a (CO_2_ concentration) the amount of noise presented on the CO_2_ signal can be easily appreciated. Consequently, the estimation of photosynthesis showed in Figure 6b is too noisy. Furthermore, it can be observed that filtering stages have improved the overall signal quality of CO_2_ concentration at Figure 6c and net photosynthesis in Figure 6d.

Figure 6 shows only the result of 1 node, but similar results were obtained for the other nodes of the network. In order to quantify how the filtered signals improved the estimation of physiological processes, a Pearson correlation between raw and filtered photosynthesis signals against radiation signal was conducted. Results presented in Table 2 suggest that the behavior of *P_n_* when it was estimated with non-filtered signals do not correspond with the radiation pattern. In contrast, a better correlation between *Rad* and the *P_n_* estimated with filtered signals was found. In Table 2, photosynthesis with the subscript *osk* is the estimated one with filtered primary signals. The *R*-value shows the correlation weight while *p*-values below 0.05 confirm the existence of correlation between signals.

### 4.5. Environmental Signals

**Figure 7.**
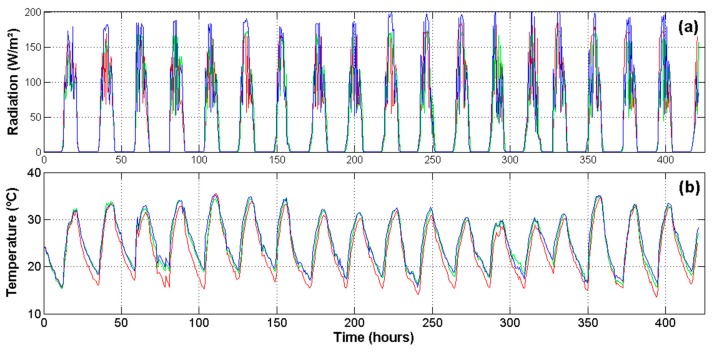

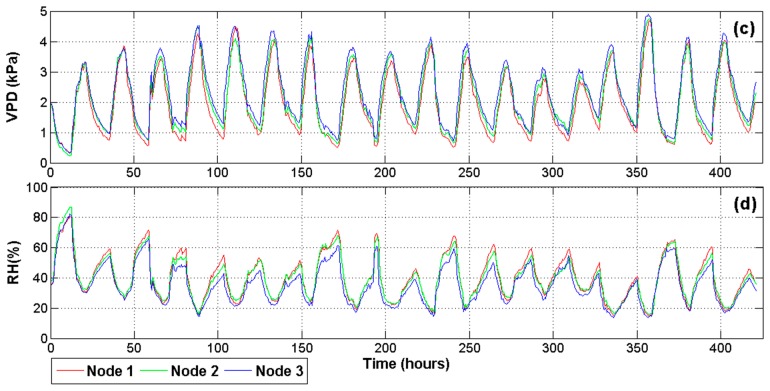
WSN environmental readings inside the greenhouse at locations of Nodes 1, 2 and 3. (**a**) Solar radiation; (**b**) air temperature; (**c**) vapor pressure deficit; and (**d**) relative humidity.

Because the experiment was carried out in a commercial greenhouse, spatial differences in the microclimate produced changes on physiological processes, even for plants undergoing the same water stress treatment. Then, it was necessary to monitor the microclimate related variables in order to understand these changes. Figure 7 illustrates the most important environmental variables monitored inside the greenhouse at three different locations. Figure 7a shows the readings for radiation during the entire experiment. This variable is noteworthy because it modifies the temperature (Figure 7b), *VPD* (Figure 7c) and *RH* (Figure 7d) of the air inside the greenhouse and therefore the transpiration rates of plants. Moreover, the photosynthesis is more sensitive to radiation than to any other factor.

**Figure 8.**
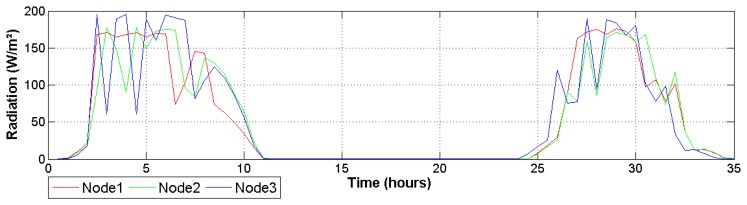
Image zooms for two days of radiation.

Differences between node readings change throughout the day, but keep a relatively regular pattern in which node 3 registers a higher temperature and drier air conditions. As can be seen in Figure 8, this behavior is influenced by the total solar radiation received by this node. This figure illustrates how the greenhouse presents spatial variations provoked mainly by the structure geometry. This is important because it helps investigators to understand abrupt changes in transpiration and photosynthetic signals estimated by the system. Finally, it is important to note that received radiation was around 175 W/m^2^, which is a suitable quantity to grow Raffaello variety of tomatoes [35].

### 4.6. Physiological Signals

**Figure 9.**
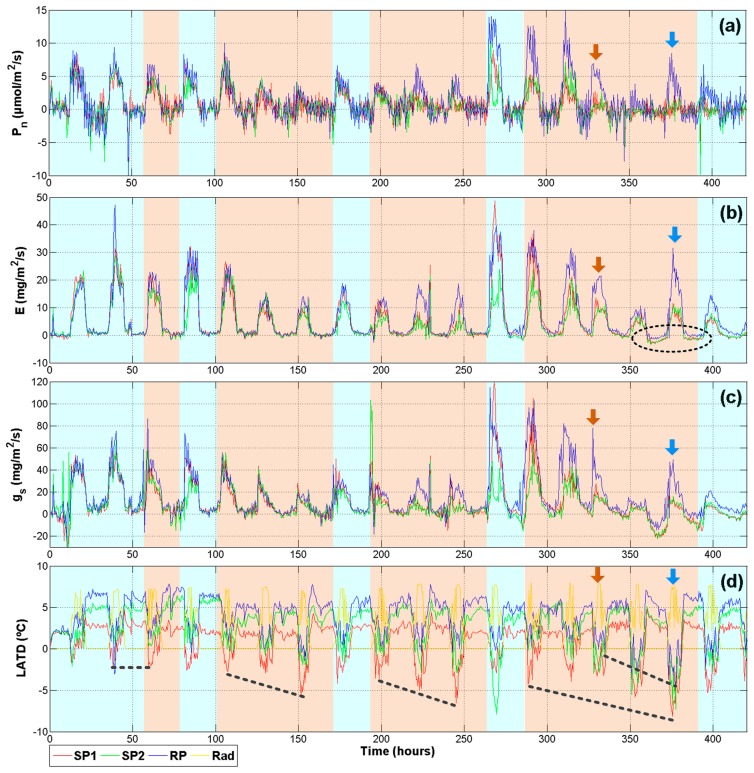
Physiological signals. (**a**) Photosynthesis; (**b**) transpiration; (**c**) stomatal conductance; and (**d**) leaf to air temperature difference.

The methodology to induce drought stress was as follows: The first two days of monitoring were a stabilizing period in which plants were watered at field capacity (10 kPa); on day three, the irrigation was suspended so that two plants could reach 30–40 kPa soil drought conditions. Then, plants were rehydrated in order to avoid reaching permanent wilting point (PWP). After one rehydration day, the drought treatment began again. The experiment lasted 19 days. As is illustrated in Figure 9, red and green signals correspond to plants suffering from drought stress (SP1 and SP2 respectively). The reference plant (RP) that was continuously irrigated at 10 kPa is represented with the blue signal. The light blue shadow indicates irrigation and light orange means water depletion. The brown arrows indicate irrigation interruption for the RP. After two days the irrigation was resumed, this is indicated by the blue arrows.

Figure 9 summarizes the physiological signals that provide more information related to plant status (*P_n_*, *E*, *g_s_*, and *LATD*). As was expected, photosynthesis is not sensitive to early stages of drought. In Figure 9a, a difference between the RP and the treatments can be noticed only after three periods of stress near hour 225. Furthermore, after the fourth period of stress around hour 400, net photosynthetic activity for SP1 and SP2 was not recovered after being rehydrated. This behavior can be explained because drought periods generate an accumulative oxidative stress in the leaves until damaging photosystem II in a permanent way by reactive oxygen species [2,36].

Transpiration results (Figure 9b) show an early response to treatments, especially in the second and third stages of stress. However, the most interesting behavior is at the end, enclosed by the dark ellipse, a low amplitude negative transpiration rate indicates that leaves are taking water vapor from the atmosphere instead of expelling it. This is a defense mechanism observed in plants under severe drought [37]. The stomatal conductance (Figure 9c) presents more marked differences as compared with *P_n_*. Even in the first day of drought, SP1 and SP2 present a sudden drop in *g_s_*. This is explained because the stomata closure and the decrease of *g_s_* are the first defenses plants employ in order to reduce the amount of water lose through the stomata and it is related more to soil drought than leaf water status [2]. The higher decrease in the third stage of drought could be related to a decrease of *g_m_* because the leaves are preparing for severe stress conditions.

The final graph (Figure 9d) illustrates the difference between air temperature and leaf temperature. Herein, a yellow line illustrates the day where the *LADT* must be zero or slightly positive. This is a normal behavior because in well-watered plants the *T_leaf_* is cooler than *T_a_*. However, if plants are under drought conditions, the *T_leaf_* is higher than *T_a_*. This tendency is clearly noticed in Figure 9d, where once the water depletion begins an increase in negative readings appears. This tendency is illustrated with the dashed black lines. Nevertheless, despite being under the same conditions, SP2 always presented a better tolerance to the stress than SP1. This can be noted because the red line presents more negative and sudden changes in *LATD*. On the other hand, the reference plant showed stable behavior with zero or positive values until the irrigation was suspended at hour 330. After this point, the drought was maintained for two days and a clear drop of blue line appears. After the rehydration day during hour 375, the *LATD* of RP slowly returns to zero and positive values.

Finally, it is important to mention that a significant reduction in height and the leaf areas of plants under drought was expressed. At the end of the experiment, plants under drought conditions maintained heights of approximately 80% of non-stressed plants.

### 4.7. Indexes and DWT Analysis

Despite the fact that Figure 9 provides important information about effects of drought on plants, the analysis requires data from at least two days in order to be able to notice a behavioral pattern. The problem with performing single day assessment is the amount of remnant noise, mainly for *P_n_* and *g_s_* signals. Another problem is the variation in growing conditions throughout the greenhouse, which may cause plants under the same treatment to respond quite differently. The variable that mainly affects the results is the solar radiation. This problem was addressed using the index previously described in Equation (1). Therefore, if plants under the same treatment receive different radiation levels, the difference in variable responds is mitigated permitting a better comparison. This methodology was useful for *P_n_*, *E*, and *g_s_* signals which presented a higher component of noise as compared with *VPD* or *LATD*.

**Figure 10.**
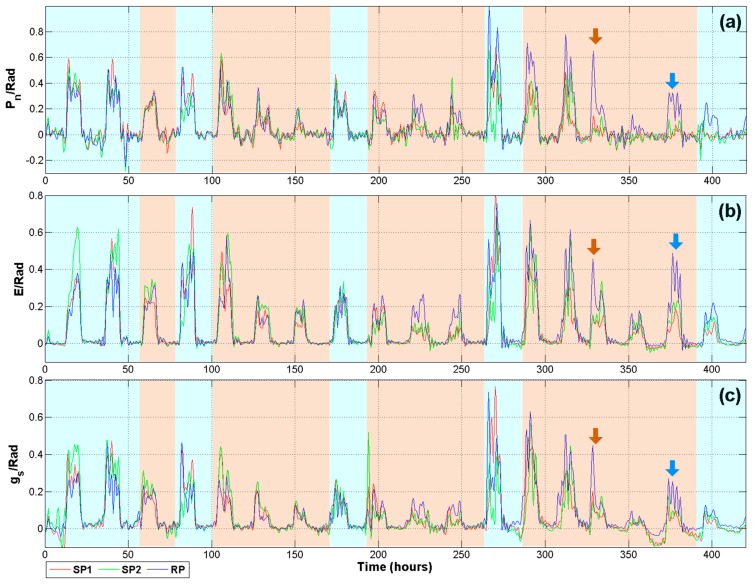
Wavelet of processes/Radiation indexes. (**a**) Photosynthesis; (**b**) transpiration; and (**c**) stomatal conductance.

As it can be seen in Figure 3, after the normalization process, these signals were analyzed using the DWT. In a preliminary experiment, several configurations were performed in order to explore the best one to extract information from signals. Finally, the DWT applied to filter the signals illustrated in Figure 10 uses a mother wavelet Daubechies db40 at a level A2 that rejects signals outside the range from 0 to 0.27 mHz bandwidth. This criterion was selected because lower mother wavelet levels discriminate important information related with abrupt changes due to radiation. Also, db40 mother wavelet required less computational resources compared with other wavelets such as Symmlets. The high frequency analysis of D levels is not reported because no clear patterns were found; this behavior could be a consequence of the system slow sampling frequency. The new version of *P_n_* signals corresponding to Figure 10a presents a considerable reduction in the high frequency noise compared to Figure 9a, where after several stages of filtering, the *P_n_* signal maintains a considerable amount of noise, this could be probably an aliasing of a frequency generated for the IRGA Sensor itself. However, after the use of DWT analysis such as an extra filtering stage, this component of noise was reduced allowing a comparison along one single day.

Furthermore, in order to avoid the removal of information related with the plant response and considering that *P_n_* is highly related with *Rad*, a Pearson correlation was conducted to compare *Rad* with *P_n_* before the DWT processing. The correlation results are presented in Table 3 to support the idea that DWT rejects the noise on photosynthesis signal but it keeps the information related with photosynthesis itself. As it can be appreciated, the correlation between *P_n_* and *Rad* slightly increases when *P_n_* is filtered with the DWT. Only node 3 did not repeat this trend. However, the decrease in the correlation is not too high. Table 3 presents the results of the hypothesis test of no correlation. The *p*-values suggest that null hypothesis is rejected.

**Figure 11.**
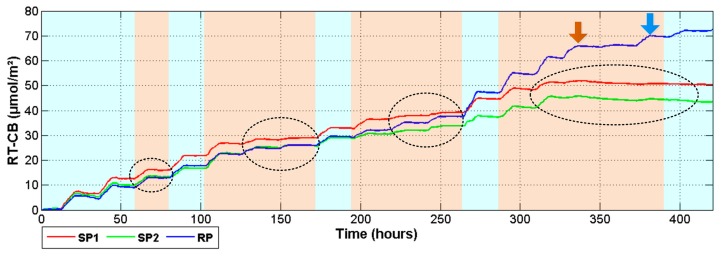
Real Time-Carbon Balance (*RT*-*CB*).

Finally, in order to understand the impact of drought for long-term development and health of the plants, the analysis of *P_n_* integral calculation which is named as Real Time-Carbon Balance index was proposed to explore the response of plants under drought conditions. As it can be seen in Figure 11, during the day *RT*-*CB* increases but stops or slightly decreases during the nights. This behavior is a result of the photosynthetic and respiration activity; but, as it is indicated with dark ellipses, when the plants were subjected to drought, the *RT*-*CB* signals remained constant until the rehydration day. Here it is important to state that the first day of drought did not significantly affected plant response and changes appeared until day two. During the rehydration days corresponding to hours 75, 175, and 275; the plants were recovered and photosynthetic activity was normalized. Nevertheless, SP1 and SP2 did not recover after the fourth period of drought, even when plants were watered at approximately hour 380. Here, the *RT*-*CB* index maintains the negative tendency which means that the photosynthetic activity stops, and the net respiration increases causing a loss of dry matter. Figure 11 also shows the exponential behavior of the photosynthesis activity as the plant grows, because around hour 275 of experimentation, the *RT*-*CB* registered an important increase for the reference plant. However, this tendency changed when the irrigation was suspended. The first day of scarcity, as indicated with the brown arrow did not change the plant response. However, the next day a fall in the Carbon assimilation was reported. This tendency continues until normal irrigation was re-established in the day marked with the blue arrow.

## 5. Conclusions

In this investigation, a smart sensor system was developed to monitor primary variables in plants. Then, this information was then used to estimate physiological processes such as photosynthesis, transpiration, and stomatal conductance. The proposed experiment demonstrates the capabilities of the system to detect stress in plants submitted to soil drought conditions. It also reveals that even under real operation conditions (greenhouse applications) the system properly estimates the aforementioned physiological processes. However, important considerations must be taken into account if the system pretends to be operated outside due to sunlight and rain conditions. But it is important to state that this is a prototype that can be improved in a future. Another central consideration relies on the leaf chamber design and stress conditions that are produced on isolated leaves. During the experiment, it was necessary to periodically changed between leaves. However, during periods of three days not important damage over the samples was appreciated. This may be due to the Nylamid-acrilic materials utilized in the leaf chamber design, which do not overheat under sunlight such as aluminum based chambers that are used in commercial devices.

In addition, the DWT was used to process the signal combined with an index that adjusts the estimation according to the plants surrounding environment. It resulted useful in order to perform a day by day comparison for drought detection, which is important because conventional analysis requires long time to detect drought conditions. Moreover, the *RT*-*CB* index provides an alternative method for monitoring plant growth without using destructive laboratory analysis. Therefore, *RT*-*CB* provides information about irregular growing circumstances such as drought. Finally, the proposed digital signal processing methodology implemented in the gas exchange system represents an alternative that can be used to detect and monitor drought under real growth conditions. Also, this methodology can be utilized for filtering purposes in precision agriculture applications where the signal-to-noise ratio is high (like chlorophyll fluorescence or impedance sensor applications). Furthermore, it can be utilized to explore time-frequency properties of different kinds of signals.

## Figures and Tables

**Table 1.** Equations for the estimation of physiological processes of plants.

**Variable**	**Equation**	**Unit of Measurement**
Mass flow rate per area	$W=(2005.39)\frac{\left(V)(P\right)}{\left(T_{a}K)(A\right)}$	mmol/m^2^/s
Photosynthesis	*P_n_* = (*W*)(*C_i_* − *C_o_*)	μmol/ m^2^/s
Transpiration	$E=(W)(1000)(18.02)\frac{\left(e_{o}-e_{i}\right)}{\left(P-e_{o}\right)}$	mg/m^2^/s
Stomatal conductance	$g_{s}=\frac{W}{\left(\frac{e_{leaf}-e_{o}}{e_{o}-e_{i}}\right)\left(\frac{P-e_{o}}{P}-r_{b}(W)\right)}(1000)$	mmol/m^2^/s
Vapor pressure deficit	*VPD* = *e_s_* − *e_i_*	kPa
Leaf to air temperature difference	*LATD* = *T_a_* − *T_leaf_*	°C

**Table 2.** Correlation analysis results for radiation against photosynthesis with and without filtering.

**Variables**	**Photosynthesis–Radiation**		**Photosynthesis_osk_–Radiation**	
		
**Coefficients**	***R***	***p***	***R***	***p***
Node 1	0.0672	0.0515	0.5146	<0.0001
Node 2	0.2023	<0.0001	0.5307	<0.0001
Node 3	0.1027	0.0028	0.6557	<0.0001

**Table 3.** Photosynthesis–radiation correlation results with and without DWT filtering.

**Variables**	**Photosynthesis_osk_–Radiation**		**Photosynthesis_DWT_–Radiation**	
		
**Coefficients**	***R***	***p***	***R***	***p***
Node 1	0.5146	<0.0001	0.5600	<0.0001
Node 2	0.5307	<0.0001	0.5574	<0.0001
Node 3	0.6557	<0.0001	0.6308	<0.0001
